# Evaluation of block-sequential regularized expectation maximization reconstruction of ^68^Ga-DOTATOC, ^18^F-fluoride, and ^11^C-acetate whole-body examinations acquired on a digital time-of-flight PET/CT scanner

**DOI:** 10.1186/s40658-020-00310-1

**Published:** 2020-06-15

**Authors:** Elin Lindström, Lars Lindsjö, Anders Sundin, Jens Sörensen, Mark Lubberink

**Affiliations:** 1 Radiology & Nuclear Medicine, Department of Surgical Sciences, Uppsala University, SE-751 85 Uppsala, Sweden; 2 Medical Physics, Uppsala University Hospital, SE-751 85 Uppsala, Sweden; 3 PET Centre, Uppsala University Hospital, SE-751 85 Uppsala, Sweden

**Keywords:** PET/CT, Regularized image reconstruction, Penalization factor, Block-sequential regularized expectation maximization, BSREM

## Abstract

**Background:**

Block-sequential regularized expectation maximization (BSREM) is a fully convergent iterative image reconstruction algorithm. We hypothesize that tracers with different distribution patterns will result in different optimal settings for the BSREM algorithm. The aim of this study was to evaluate the image quality with variations in the applied *β*-value and acquisition time for three positron emission tomography (PET) tracers. NEMA image quality phantom measurements and **c**linical whole-body digital time-of-flight (TOF) PET/computed tomography (CT) examinations with ^68^Ga-DOTATOC (*n* = 13), ^18^F-fluoride (*n* = 10), and ^11^C-acetate (*n* = 13) were included. Each scan was reconstructed using BSREM with *β*-values of 133, 267, 400, and 533, and ordered subsets expectation maximization (OSEM; 3 iterations, 16 subsets, and 5-mm Gaussian post-processing filter). Both reconstruction methods included TOF and point spread function (PSF) recovery. Quantitative measures of noise, signal-to-noise ratio (SNR), and signal-to-background ratio (SBR) were analysed for various acquisition times per bed position (bp).

**Results:**

The highest *β*-value resulted in the lowest level of noise, which in turn resulted in the highest SNR and lowest SBR. Noise levels equal to or lower than those of OSEM were found with *β*-values equal to or higher than 400, 533, and 267 for ^68^Ga-DOTATOC, ^18^F-fluoride, and ^11^C-acetate, respectively. The specified *β*-ranges resulted in increased SNR at a minimum of 25% (*P* < 0.0001) and SBR at a maximum of 23% (*P* < 0.0001) as compared to OSEM. At a reduced acquisition time by 25% for ^68^Ga-DOTATOC and ^18^F-fluoride, and 67% for ^11^C-acetate, BSREM with *β*-values equal to or higher than 533 resulted in noise equal to or lower than that of OSEM at full acquisition duration (2 min/bp for ^68^Ga-DOTATOC and ^18^F-fluoride, 3 min/bp for ^11^C-acetate). The reduced acquisition time with *β* 533 resulted in increased SNR (16–26%, *P* < 0.003) and SBR (6–18%, *P* < 0.0001 (*P* = 0.07 for ^11^C-acetate)) compared to the full acquisition OSEM.

**Conclusions:**

Within tracer-specific ranges of *β*-values, BSREM reconstruction resulted in increased SNR and SBR with respect to conventional OSEM reconstruction. Similar SNR, SBR, and noise levels could be attained with BSREM at relatively shorter acquisition times or, alternatively, lower administered dosages, compared to those attained with OSEM.

## Background

PET images are usually reconstructed by the clinical standard-of-care method ordered subsets expectation maximization (OSEM). A known shortcoming of maximum likelihood reconstruction, methods like OSEM, is excessive noise in the images if too many iterations are performed, and insufficient convergence associated with underestimated radioactivity concentrations especially in small lesions and those that are obscured by larger areas of uptake when too few iterations are performed. Regularization is commonly achieved by terminating the iterative process prior to convergence, but the recently developed regularizing technique instead utilizes a penalty function that prohibits excessive noise. The block-sequential regularized expectation maximization (BSREM) algorithm (Q.Clear; GE Healthcare) incorporates a relative difference penalty that balances the properties of a quadratic and non-quadratic penalty, i.e. spatial smoothness and preservation of edges [1–4]. The algorithm allows every single image voxel to achieve full convergence [3, 5]. Hence, in contrast to OSEM, BSREM offers only one controlling reconstruction parameter that is available to its users, a global regularization parameter denoted *β* [4].

In a previous study evaluating ^18^F-FDG on the same digital time-of-flight (TOF) PET/CT scanner, BSREM was found to provide higher maximum standardized uptake value (SUV_max)_ values and improved signal-to-noise ratio (SNR) and signal-to-background ratio (SBR) compared to OSEM at matched levels of noise [6]. The clinical benefit of the algorithm for ^18^F-FDG PET using other PET/CT scanner models has previously been reported [8–12]. Also, penalized reconstruction of ^90^Y-PET data acquired on a conventional Discovery 710 PET/CT scanner resulted in a higher contrast-to-noise ratio than OSEM and penalized reconstruction images received higher scores by the radiologists [8]. Additionally, an ^18^F-fluoride PET/CT study, performed on a Discovery 690 scanner, showed that image acquisition of 1 min/bp and BSREM resulted in similar quality as 3 min/bp images reconstructed with standard methods [9].

In the present study, we investigated the behaviour of BSREM in relation to OSEM regarding image quality in terms of noise, SNR, and SBR, with three non-^18^F-FDG PET tracers regularly used in the clinical practice; gallium-68-DOTA-D-Phe^1^-Tyr^3^-octreotide (^68^Ga-DOTATOC), carbon-11-acetate (^11^C-acetate), and ^18^F-fluoride. ^68^Ga-DOTATOC is a radiolabelled somatostatin analogue used for imaging of neuroendocrine tumours [13–15]. ^11^C-acetate, similarly to ^11^C- and ^18^F-choline, can be used for imaging of the cell membrane lipid metabolism that is typically increased in prostate cancer cells [16–20], and ^18^F-fluoride is used for bone imaging, especially for the detection and monitoring of bone metastases in prostate cancer [21, 22]. These three tracers were chosen for evaluation because they are used in the clinical routine and exhibit characteristic uptake patterns that are clearly different from the typical FDG distribution, ranging from very specific uptake in bone lesions combined with very low uptake in soft tissues for ^18^F-fluoride, high lesion uptake accompanied by high uptake in healthy organs such as liver and kidney but virtually no signal elsewhere for ^68^Ga-DOTATOC, to a more or less homogeneous distribution pattern for ^11^C-acetate. We hypothesized that these varying tracer distribution patterns will require different settings for the BSREM algorithm to achieve optimum image quality in terms of noise, SNR, and SBR. The purpose of the study was to evaluate the image quality with variations in the applied *β*-value and acquisition time.

## Materials and methods

All patient data analyses were performed on anonymized PET/CT data collected from existing records and the reconstructed images were not used for clinical image reading. The study was approved by the regional Ethical Review Authority (Dnr, 2019-00092).

### Imaging protocol

A National Electrical Manufacturers Association (NEMA) image quality phantom experiment with ^68^Ga was performed and compared to the results from a previous study with ^18^F acquired using similar settings [6, 7]. The phantom background region and spheres were filled with activity concentrations of, respectively, 5.05 kBq/ml and 21.78 kBq/ml of ^68^Ga, and 2.69 kBq/ml and 10.77 kBq/ml of ^18^F, yielding a 4.3:1 and 4.0:1 sphere-to-background concentration ratio. The phantom was scanned until 100 million prompt counts were acquired on both occasions.

Clinical whole-body PET/CT examinations with ^68^Ga-DOTATOC (*n* = 13), ^18^F-fluoride (*n* = 10), and ^11^C-acetate (*n* = 13) were analysed. The ^68^Ga-DOTATOC cohort included patients with small intestinal neuroendocrine tumours (NET), lung-NET, rectal-NET, gastric-NET, pancreatic-NET, carcinoma of unknown primary, and medullary thyroid cancer diagnosis. The ^18^F-fluoride and ^11^C-acetate cohorts both included patients with prostate cancer and verified bone metastases, and relapse after primary treatment such as radical prostatectomy or radiation therapy. All examinations were acquired in 3-dimensional mode using a digital TOF Discovery MI PET/CT (GE Healthcare) with a 4-ring setup, providing axial and transaxial FOV of 20 and 70 cm, respectively. Further scanner specifications include lutetium yttrium oxyorthosilicate detectors coupled to silicon photomultipliers, a timing resolution of circa 370 ps, a sensitivity of 14 cps/kBq, and an intrinsic spatial resolution of approximately 4.0 mm at the centre of the FOV [23]. The PET/CT examinations were performed with an acquisition time of 3 min/bp (^11^C-acetate) or 2 min/bp (^68^Ga-DOTATOC, ^18^F-fluoride) from the mid-thigh to the base of the skull.

Images were reconstructed by using OSEM with 3 iterations, 16 subsets, and a 5-mm Gaussian post-processing filter, and BSREM with *β*-values of 133, 267, 400, and 533. TOF and point spread function (PSF) were applied with both reconstruction methods. A matrix size of 256 × 256 was used, resulting in a voxel size of 2.73 × 2.73 × 2.79 mm^3^. Furthermore, list-mode files were re-binned by using the first 2 and 1 min/bp (^11^C-acetate) or 1.5 and 1 min/bp (^68^Ga-DOTATOC, ^18^F-fluoride) of the acquired data and reconstructed using the abovementioned settings.

### Image analysis

NEMA image quality phantom results were analysed using in-house-developed software in Matlab (MathWorks) as previously described [6, 7]. Background variability and contrast recovery were calculated according to the NEMA standards protocol [7].

Analyses of clinical data were performed on an Advantage Workstation (Volume Viewer 4.7 ext. 8; GE Healthcare) with the PET/CT Review software. Lesion metrics were derived using a 3-dimensional segmentation at 41% of the maximum voxel value and reference values were obtained from a spherical volume of interest (approximately 3 cm in diameter) placed in the right lobe of the liver with visually confirmed uniform uptake. The lesion delineations were performed in the OSEM reconstruction of each examination and thereafter the location of the maximum voxel value was transferred to the respective BSREM reconstructions, where new volumes of interest were redefined at a 41% threshold. The total number of lesions included in the analysis was 38 with ^68^Ga-DOTATOC, 33 with ^18^F-fluoride, and 36 with ^11^C-acetate. Level of noise was measured in the liver tissue and calculated as standard deviation (SD) divided by the mean standardized uptake value (SUV_mean_) of the liver reference sphere (Eq. 1). SNR was defined as the lesion SUV_max_ to noise level ratio and SBR as lesion SUV_max_ to liver reference SUV_mean_ ratio (Eqs. 2 and 3). The optimal *β*-value was defined as the value that yielded noise equivalent to that of OSEM.

1 $$ \mathrm{Noise}=\frac{\mathrm{S}{\mathrm{D}}_{\mathrm{reference}}}{\mathrm{S}\mathrm{U}{\mathrm{V}}_{\mathrm{mea}{\mathrm{n}}_{\mathrm{reference}}}} $$

2 $$ \mathrm{SNR}=\frac{\mathrm{SU}{\mathrm{V}}_{\mathrm{max}}}{\mathrm{Noise}} $$

3 $$ \mathrm{SBR}=\frac{\mathrm{SU}{\mathrm{V}}_{\mathrm{max}}}{\mathrm{SU}{\mathrm{V}}_{\mathrm{mea}{\mathrm{n}}_{\mathrm{reference}}}} $$

### Statistical analysis

Values are presented as mean ± SD. A paired non-parametric two-tailed *t* test (Wilcoxon’s signed rank test) was used when comparing BSREM to OSEM with considered statistical significance for *P* values less than 0.05. Spearman rank correlation (*r*) was used to determine the degree of association between relative difference in SUV and lesion volume.

## Results

### NEMA image quality phantom

**Fig. 1:**
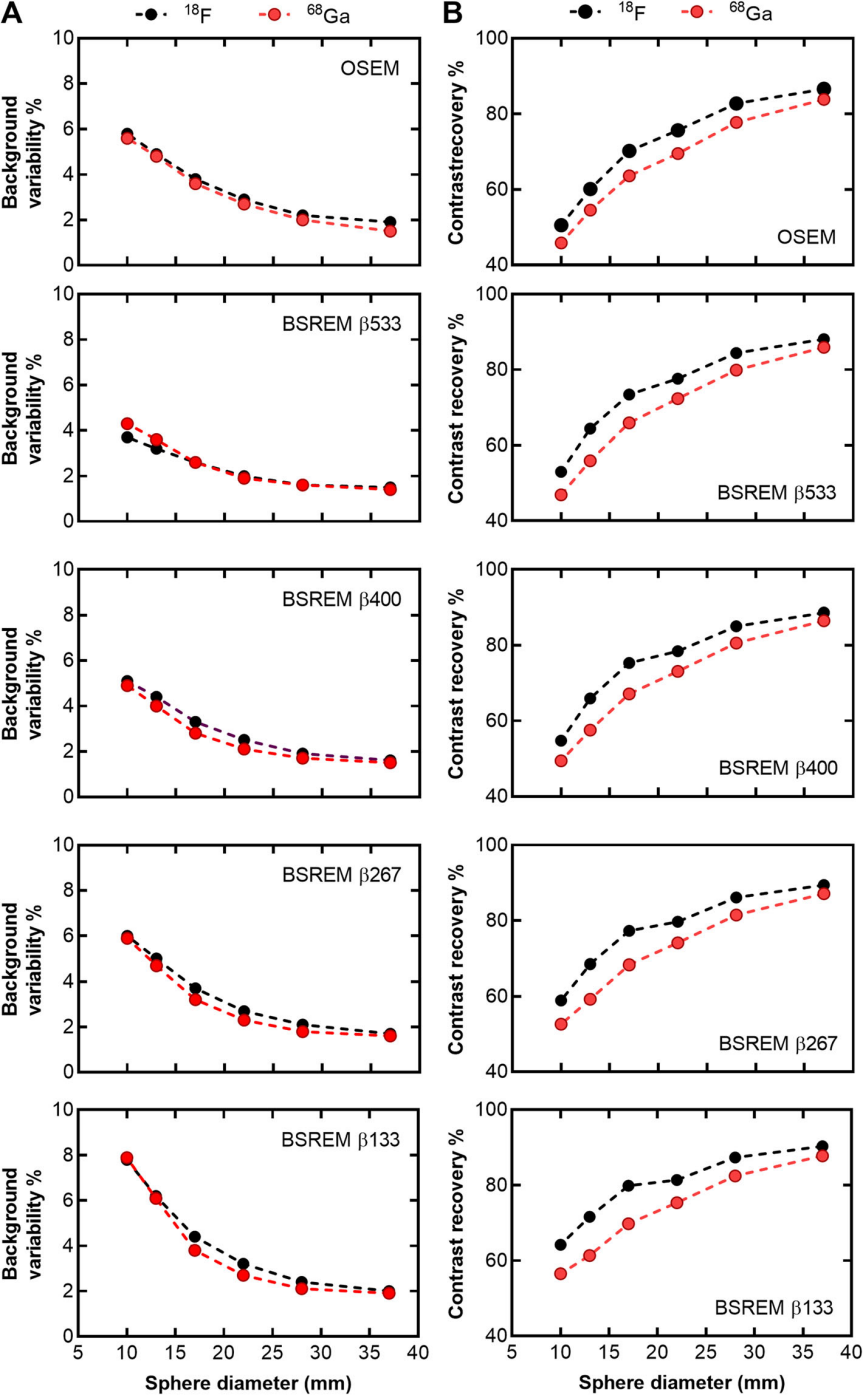
Background variability % (**a**) and contrast recovery % (**b**) of NEMA image quality phantom measurements with ^18^F (black) and ^68^Ga (red). The five graphs in each column display the results of separate reconstruction methods; OSEM (3 iterations, 16 subsets, 5-mm Gaussian post-processing filter) and BSREM with *β*-values of 133, 267, 400, and 533. Both reconstruction methods included TOF and PSF recovery

NEMA phantom results are displayed in Fig. 1. Background variability was similar for ^18^F and ^68^Ga for all reconstruction methods and sphere sizes. The background variability increased with decreasing *β*-value. In contrast recovery, there was a consistent difference between ^18^F and ^68^Ga for all reconstruction methods and sphere sizes with a slightly lower contrast recovery for ^68^Ga than ^18^F.

### ^68^Ga-DOTATOC

**Fig. 2:**
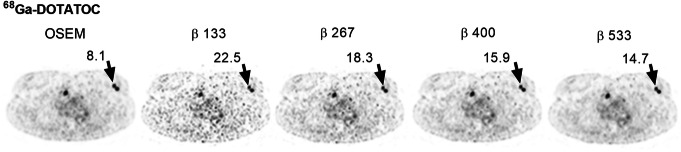
Transversal ^68^Ga-DOTATOC PET images of a patient with small intestinal neuroendocrine metastases. Images were acquired 72 min post-injection and reconstructed with 2 min/bp using OSEM (3 iterations, 16 subsets, 5-mm Gaussian post-processing filter) and BSREM with *β*-values of 133, 267, 400, and 533. Both reconstruction methods included TOF and PSF recovery. The administered activity was 1.8 MBqkg^−*1*^. The SUV_max_ of the lesion (arrow) is shown for the respective reconstruction

**Fig. 3:**
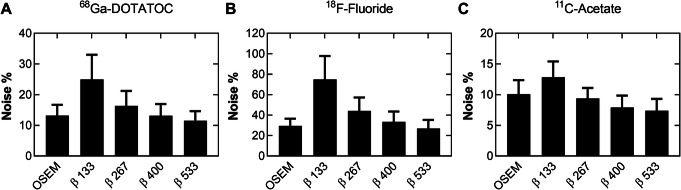
Noise measured in normal liver tissue for ^68^Ga-DOTATOC (**a**), ^18^F-fluoride (**b**), and ^11^C-acetate (**c**), respectively, using BSREM and OSEM (3 iterations, 16 subsets, 5-mm Gaussian post-processing filter). Both reconstruction methods included TOF and PSF recovery

**Fig. 4:**
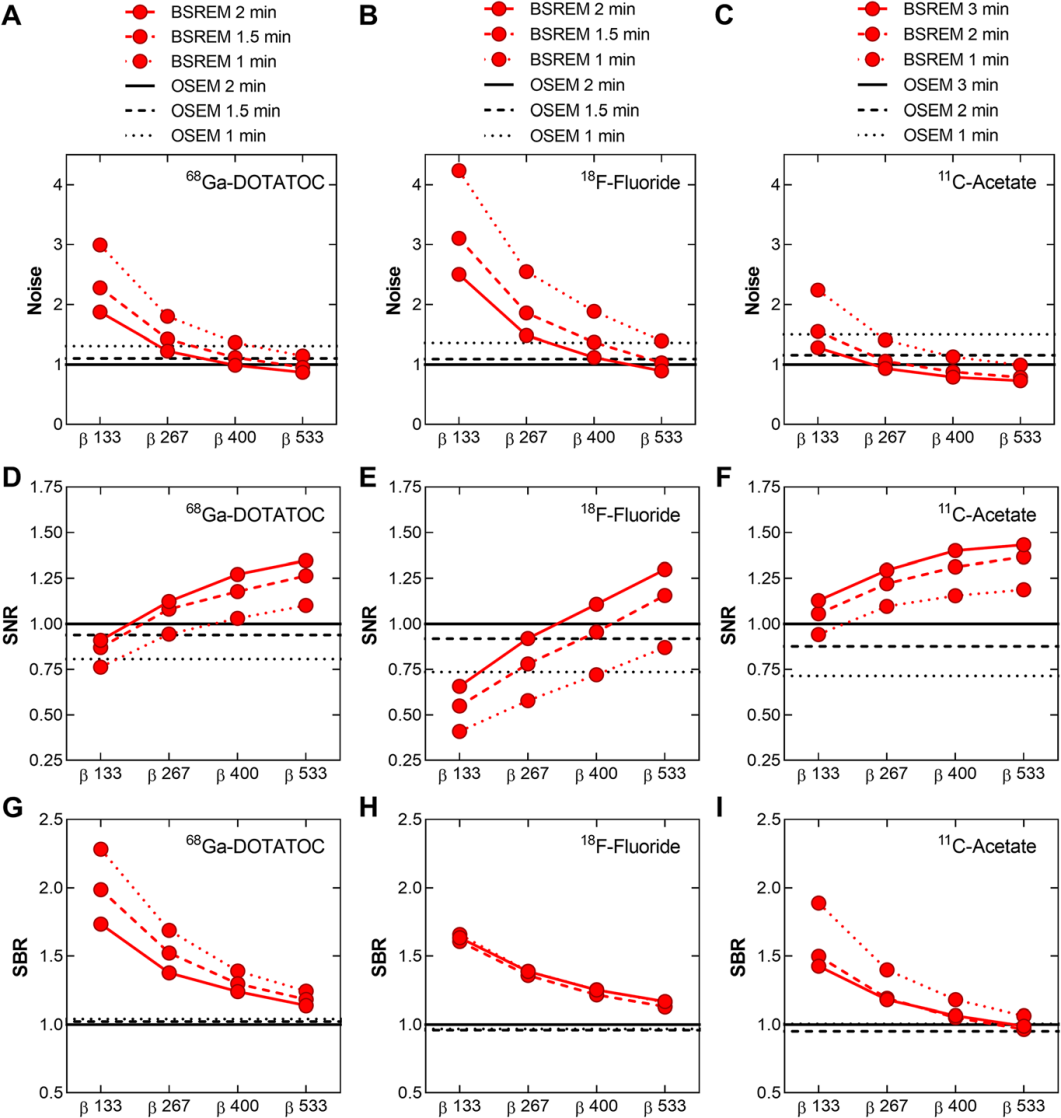
Mean noise in normal liver tissue plotted against *β*-value (**a**–**c**), mean SNR against *β*-value (**d**–**f**), and mean SBR against *β*-value (**g**–**i**) for ^68^Ga-DOTATOC, ^18^F-fluoride, and ^11^C-acetate, respectively, using BSREM and OSEM (3 iterations, 16 subsets, 5-mm Gaussian post-processing filter) for three various image acquisition times per bed position (solid, dashed, and dotted lines). Both reconstruction methods included TOF and PSF recovery. All data were normalized to those obtained by OSEM at full image acquisition time

**Fig. 5:**
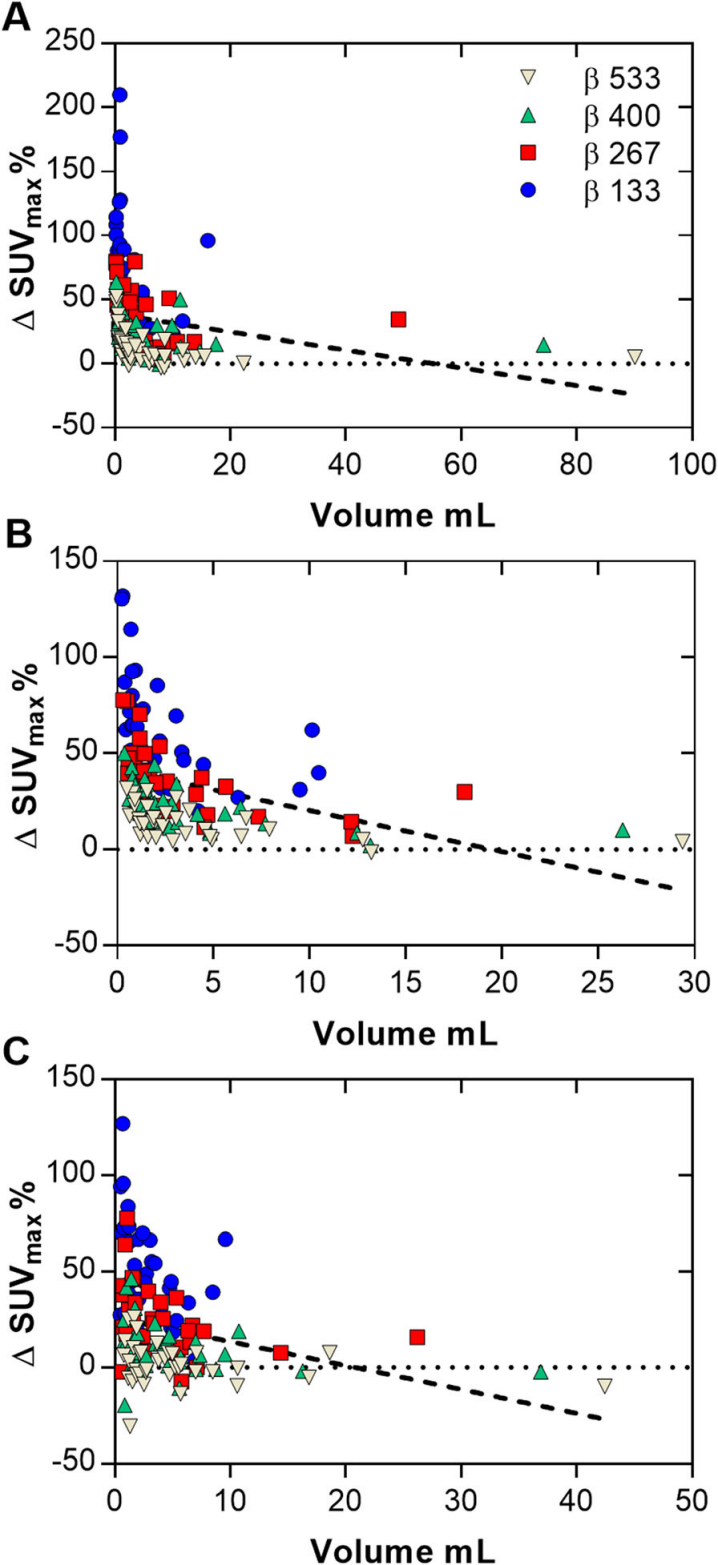
Plots showing the relative increase of SUV_max_ in lesions for BSREM with *β*-values of 133, 267, 400, and 533 compared with OSEM (3 iterations, 16 subsets, 5-mm Gaussian post-processing filter) over lesion volume, for ^68^Ga-DOTATOC (**a**), ^18^F-fluoride (**b**), and ^*11*^C-acetate (**c**). Both reconstruction methods included TOF and PSF recovery. Dashed lines indicate the linear regression of the respective cohort (for all *β*-values)

An example of a ^68^Ga-DOTATOC PET scan is shown in Fig. 2, illustrating the effect of different reconstruction methods. The injected activity was 2.3 ± 0.4 MBqkg^−1^ (range, 1.8–2.8 MBqkg^−1^) of ^68^Ga-DOTATOC and the uptake time before examination was 62 ± 4 min (range, 59–72 min). The mean liver reference volume was 18.5 ± 0.8 mL (range, 16.8–19.6 mL) and the SUV_mean_ 6.2 ± 1.5 (range 3.0–8.0) with OSEM (Table 1). The mean lesion volume was 7.6 ± 15.2 mL (range, 0.5–95.2 mL). Noise equivalence to OSEM was found with *β* 400 (− 1%, *P* > 0.99) with a resulting significant increase of SUV_max,_ SNR, and SBR by 23%, 25%, and 23%, respectively (*P* < 0.0001) (Figs. 3a and 4a, d, g). There was a negative correlation between relative difference in SUV_max_ and lesion volume when comparing BSREM with OSEM (*r* = − 0.6 measured over the entire cohort). The difference was greater in small lesion sizes, and for volumes larger than approximately 3 mL the relative difference in SUV_max_ did not change with volume (using *β* 400, *r* = − 0.8, − 0.1, respectively) (Fig. 5a).

**Table 1:** Quantitative measures of reference sphere in normal liver tissue for ^68^Ga-DOTATOC, ^18^F-fluoride, and ^11^C-acetate

Measure	OSEM	BSREM			
		*β* 133	*β* 267	*β* 400	*β* 533
^68^Ga-DOTATOC					
Volume mL	18.5 (16.8–19.6)				
SUV_max_	8.7 (5.1–11.4)	13.5 (9.6–20.1)	9.9 (6.2–13.8)	8.8 (5.1–12.0)	8.3 (4.5–11.5)
SUV_mean_	6.2 (3.0–8.0)	6.2 (3.0–8.1)	6.2 (3.0–8.1)	6.2 (3.0–8.1)	6.2 (3.0–8.1)
P^*^		> 0.7	> 0.8	> 0.9	> 0.9
SUV_SD_	0.8 (0.4–1.1)	1.5 (0.8–2.0)	1.0 (0.5–1.4)	0.8 (0.4–1.1)	0.7 (0.3–1.0)
Noise^†^	0.13 (0.07–0.21)	0.25 (0.14–0.44)	0.16 (0.09–0.27)	0.13 (0.08–0.21)	0.12 (0.07–0.18)
^18^F-Fluoride					
Volume mL	18.5 (16.5–19.6)				
SUV_max_	1.0 (0.6–1.3)	3.0 (1.4–6.7)	1.7 (0.8–3.6)	1.1 (0.6–1.5)	0.9 (0.5–1.3)
SUV_mean_	0.4 (0.3–0.7)	0.4 (0.3–0.7)	0.4 (0.3–0.7)	0.4 (0.3–0.7)	0.4 (0.3–0.7)
P^*^		> 0.7	> 0.3	> 0.2	> 0.3
SUV_SD_	0.1 (0.1–0.2)	0.3 (0.2–0.5)	0.2 (0.1–0.3)	0.1 (0.1–0.2)	0.1 (0.1–0.2)
Noise^†^	0.30 (0.22–0.40)	0.32 (0.22–0.49)	0.31 (0.16–0.49)	0.33 (0.23–0.49)	0.30 (0.16–0.49)
^11^C-Acetate					
Volume mL	18.7 (18.0–19.6)				
SUV_max_	5.7 (2.5–11.2)	6.3 (2.6–11.3)	5.6 (2.3–10.7)	5.3 (2.2–10.5)	5.2 (2.2–10.3)
SUV_mean_	4.3 (1.8–8.2)	4.3 (1.8–8.1)	4.3 (1.8–8.1)	4.3 (1.8–8.1)	4.3 (1.8–8.1)
P^*^		> 0.5	> 0.1	> 0.1	> 0.05
SUV_SD_	0.4 (0.2–1.0)	0.5 (0.3–1.1)	0.4 (0.2–0.9)	0.3 (0.2–0.8)	0.3 (0.1–0.8)
Noise^†^	0.10 (0.07–0.14)	0.13 (0.09–0.16)	0.09 (0.07–0.12)	0.08 (0.05–0.11)	0.07 (0.05–0.11)

†Noise was defined as SUV_SD_ divided by SUV_mean_

^*^P: Wilcoxon’s signed rank test, for BSREM to OSEM on SUV_mean_

*BSREM* block-sequential regularized expectation maximization, *OSEM* ordered subsets expectation maximization, *SUV* standardized uptake value, *SD* standard deviation

Reconstruction methods were OSEM (3 iterations, 16 subsets, 5-mm Gaussian post-processing filter) and BSREM with *β*-values of 133, 267, 400, and 533, and 2 min/bp. Both reconstruction methods include TOF and PSF recovery. Values represent mean (range)

The noise level increased with a shorter acquisition; with BSREM *β* 400 and 1.5 min/bp, the noise was 12% (*P* = 0.002) higher than that of OSEM at full acquisition (2 min/bp). However, using *β* 533 (1.5 min/bp) reduced the noise below that of OSEM (2 min/bp) (− 4%, *P* = 0.1) while SUV_max_, SNR, and SBR increased by 20%, 26%, and 18%, respectively (*P* < 0.0001) (Fig. 4a, d, g). At a 50% reduction in scan duration, *β* 400 (1 min/bp) resulted in similar SNR as compared to OSEM at full acquisition (2 min/bp) (3%, *P* = 0.5), while maintaining a higher level of SBR (Table 2, Fig. 4d, g). It should be noted that any chosen *β*-value in this study led to a higher SBR than OSEM irrespective of scan duration.

**Table 2:** Summary of BSREM *β*-values that resulted in similar image quality measure, either noise or SNR, as full scan duration OSEM

Image quality parameter	Scan duration (% of OSEM full scan duration)	BSREM *β*-value		
		^68^Ga-DOTATOC	^18^F-fluoride	^11^C-acetate
Noise^*^	100%	400	533	267
Noise	50%	> 533	> 533	400
SNR	100%	200	300	< 133
SNR	50%	400	> 533	133

^*^Noise was defined as SUV_SD_ divided by SUV_mean_

*BSREM* block-sequential regularized expectation maximization, *OSEM* ordered subsets expectation maximization, *SNR* signal-to-noise ratio

### ^18^F-fluoride

**Fig. 6:**
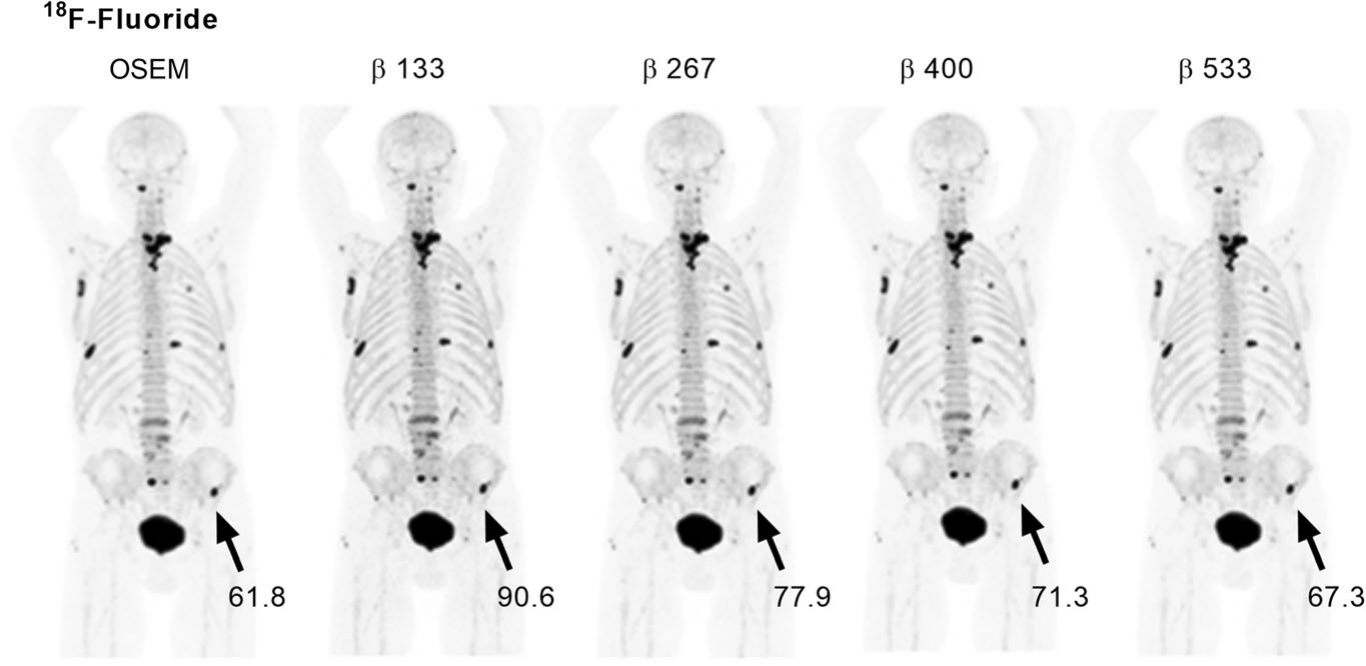
Maximum intensity projection whole-body ^18^F-fluoride PET images of a patient with prostate cancer bone metastases. Images were acquired 61 min post-injection and reconstructed with 2 min/bp using OSEM (3 iterations, 16 subsets, 5-mm Gaussian post-processing filter) and BSREM with *β*-values of 133, 267, 400, and 533. Both reconstruction methods included TOF and PSF recovery. The administered activity was 3.0 MBqkg^−*1*^. The SUV_max_ of the lesion (arrow) is shown for the respective reconstruction

As shown by the ^18^F-fluoride PET example in Fig. 6, the different reconstruction methods resulted in merely minor visual variations. The injected activity of ^18^F-fluoride was 3.0 ± 0.1 MBqkg^−1^ (range, 2.9–3.2 MBqkg^−1^) and the uptake time was 64 ± 3 min (range, 59–70 min). The mean liver reference volume was 18.5 ± 1.1 mL (range, 16.5–19.6 mL) and SUV_mean_ 0.4 ± 0.1 (range, 0.3–0.7) using OSEM (Table 1). The mean lesion volume was 4.3 ± 5.3 mL (range, 0.6–28.9 mL) with SUV_max_ 40.4 ± 24.5 (range, 13.7–108.4). A noise level equivalent to OSEM was found to lie between *β* 400 and *β* 533, with a scan duration of 2 min/bp for both reconstruction methods. *β* 400 resulted in a significantly increased level of noise whereas *β* 533 resulted in significantly lower noise than OSEM (12%, *P* = 0.0002 and − 10%, *P* = 0.0039, respectively). Both *β*-values resulting in an increased SUV_max_, SNR, and SBR by 11–30% (*P* < 0.002) as compared to OSEM (Figs. 3b and 4b, e, h). A negative correlation (*r* = − 0.6) was found between relative percentage difference in SUV_max_ (comparing BSREM with OSEM) and lesion volume when including the entire cohort. The difference was greater in small lesion sizes with a mean increase of SUV_max_ by 27% for sizes less than 3 mL and 18% for sizes larger than 3 mL with BSREM *β* 400 versus OSEM (*r* = − 0.6, − 0.7, respectively) (Fig. 5b).

Reducing image acquisition time by 25% (30 sec) to 1.5 min/bp resulted in a 3% (*P* = 0.9) increase of noise using *β* 533 compared to OSEM at full acquisition duration (2 min/bp). Despite the shorter acquisition time, SUV_max_, SNR, and SBR still improved by 17%, 16%, and 13%, respectively (*P* < 0.002), compared to OSEM at full acquisition duration (Fig. 4b, e, h). Reducing the *β*-value from 533 to 400 (1.5 min/bp) resulted in similar SNR as compared to OSEM at full acquisition (2 min/bp) (− 4%, *P* = 0.2), with a sustained higher level of SBR (Fig. 4e, h). With the limited range of *β*-values tested in this study, it was no longer possible to reach a noise level or SNR similar to OSEM (2 min/bp) when the acquisition time was further shortened by another 25% using BSREM (1 min/bp). The *β*-values that resulted in noise or SNR equivalence to OSEM at different scan durations are summarized in Table 2. Any chosen *β*-value in this study resulted in a higher SBR than OSEM irrespective of scan duration.

### ^11^C-acetate

**Fig. 7:**
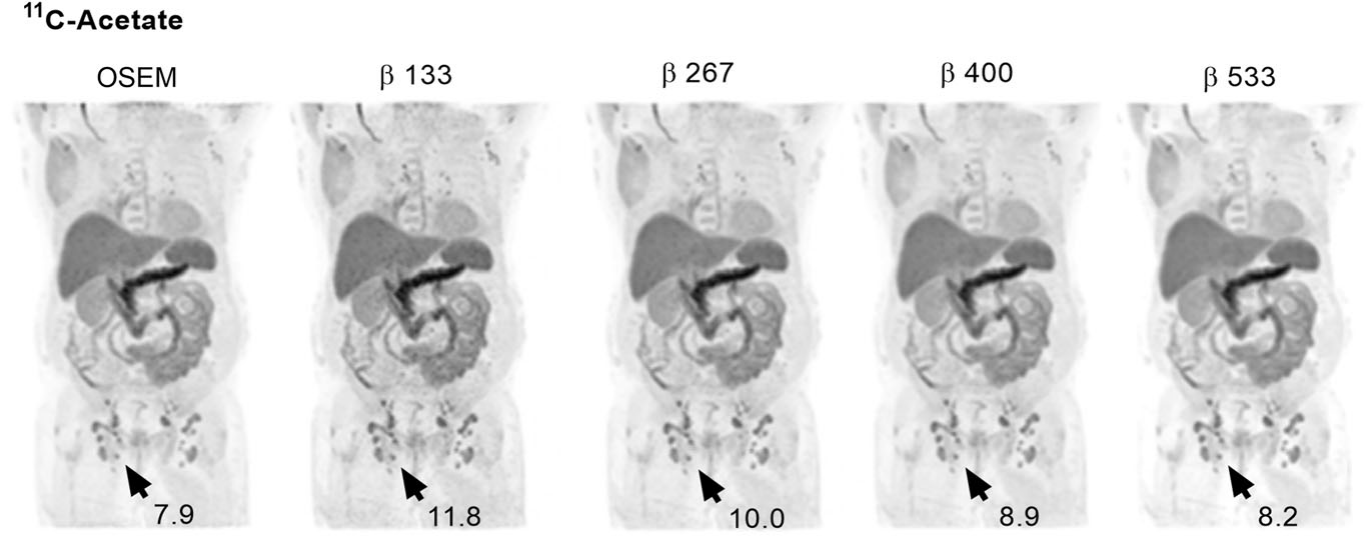
Coronal whole-body ^11^C-acetate PET images of a patient with soft tissue prostate cancer metastases. Images were acquired 11 min post-injection and reconstructed with 3 min/bed using OSEM (3 iterations, 16 subsets, 5-mm Gaussian post-processing filter) and BSREM with *β*-values of 133, 267, 400, and 533. Both reconstruction methods included TOF and PSF recovery. The administered activity was 4.2 MBqkg^−*1*^. The SUV_max_ of the lesion (arrow) is shown for the respective reconstruction.

BSREM clearly improved the image contrast and sharpness in the ^11^C-acetate PET images as compared with OSEM (Fig. 7). The injected activity was 4.8 ± 0.3 MBqkg^−1^ (range, 4.2–5.4 MBqkg^−1^) of ^11^C-acetate and the uptake time was 11 ± 1 min (range, 10–13 min). The mean liver reference volume was 18.7 ± 0.6 mL (range, 18.0–19.6 mL) and the SUV_mean_ was 4.4 ± 1.8 (range, 1.8–8.2) measured in OSEM images (Table 1). Mean lesion volume was 4.4 ± 4.7 mL (range, 0.7–22.7 mL) with mean SUV_max_ 7.7 ± 2.1 (range, 4.0–12.2). Noise levels closest to those of OSEM were found with *β* 267 (− 6%, *P* = 0.02) with a resulting significant increase of SUV_max_, SNR, and SBR by 23%, 32% and 23%, respectively (*P* < 0.0001) (Figs. 3c and 4c, f, i). There was a slight negative correlation (*r* = − 0.4) measured over the entire cohort between relative percentage difference in SUV_max_ and lesion volume comparing BSREM with OSEM. The increase of SUV_max_ using *β* 267 was 28% for lesions of sizes less than 3 mL and 17% for lesions larger than 3 mL (*r* = − 0.3, − 0.5, respectively) (Fig. 5c).

At a reduced acquisition time, BSREM *β* 267 (2 min/bp) remained closest to OSEM (3 min/bp) in terms of noise (6%, *P* = 0.3), with an increase of SUV_max_, SNR, and SBR by 25%, 22%, and 19%, respectively (*P* < 0.0001) (Fig. 4c, f, i). Using 1 min/bp resulted in *β* 533 being noise equivalent (− 1%, *P* = 0.9) to OSEM (3 min/bp) with a resulting increase of SUV_max_, SNR, and SBR by 12%, 19%, and 6%, respectively (*P* < 0.003 except for SBR *P* = 0.07). At a 67% reduction in scan duration, an estimated *β* 200 (1 min/bp) resulted in similar SNR as compared to OSEM at full acquisition (3 min/bp) (Fig. 4f). Also, the estimated *β* 200 (1 min/bp) resulted in a sustained higher SBR as compared to OSEM (3 min/bp) (Fig. 4i).

## Discussion

The objective of this study was to evaluate the relationship between BSREM and OSEM reconstruction regarding image quality with variations in the applied *β*-value and acquisition time, as well as investigate a potential tracer dependency of the respective *β*-values. Image quality was measured in terms of noise in liver tissue, SNR, and SBR. The results showed that the BSREM reconstruction algorithm allows for shorter acquisition time and/or less injected activity, as compared with OSEM, to achieve similar image quality. Prior research has demonstrated comparable results for ^18^F-FDG PET image reconstruction [24–28]. For referencing, a comparison with ^18^F-FDG is shown in Supplemental Figs. S1–3 using data from a previous study applying the same methodology [6].

The *β*-value for reaching noise equivalence to OSEM was found to be tracer dependent. The count statistics and uptake pattern vary between the tracers tested in this study and are likely the main reasons for the different optimal regularization parameters. Low-contrast tracers, like ^11^C-acetate, seem to require a lower *β*-value than sharp contrast tracers, like ^18^F-fluoride, to reach a noise level equivalent to that of OSEM. The soft tissue background uptake was low in the ^18^F-fluoride images (SUV_mean_ range, 0.3–0.7), while the lesions had a substantially higher uptake; hence, the visual effect of the BSREM reconstruction was not as prominent as for the two other tracers assessed in the study. The quantitative measures were, however, significantly different to those obtained using OSEM. PET with ^11^C-acetate resulted in rather homogeneous uptake with a low image contrast as a consequence, allowing for a lower applied *β*-value without compromising the image quality in terms of increased noise, whereas ^68^Ga-DOTATOC resulted in a similar relation between quantitative measures based on OSEM and BSREM as for ^18^F-FDG (Supplemental Figure S1). Apart from the count statistics and uptake pattern variation between the tracers likely being the main reasons for the different optimal regularization parameters, the positron kinetic energy is another contributing factor. The positron range in tissue adds to the partial volume effect that will limit the image spatial resolution, and consequently the overall image quality, thus the positron range may also influence the optimal *β*-value. In the present study, comparison between ^18^F and ^68^Ga results obtained from NEMA image quality phantom testing showed similar background variability but slightly lower contrast recovery for ^68^Ga than ^18^F [6]. Although some of the difference between the recovery for ^18^F and ^68^Ga may be due to experimental uncertainty, it is likely that the lower image resolution of ^68^Ga, due to its higher positron range, is the main cause of the difference. However, the results clearly show that the choice of *β*-value does not affect differences between ^68^Ga and ^18^F since the differences are consistent across *β*-values.

Supported by studies previously addressing BSREM image reconstruction for ^18^F-FDG and ^68^Ga-PSMA, it should be mentioned that a numerical equivalence in noise between the two different types of algorithms does not entail similar visual appearance nor detectability [6, 29]. The sample size was sufficient in order to demonstrate the differences between the two assessed reconstruction techniques and varying reconstruction and acquisition parameters. Another limiting factor was the short range and low number of tested *β*-values. Including higher *β*-values in the analysis would probably have allowed for reaching a noise level similar to that of OSEM with 2 min/bp using BSREM with 1 min/bp for ^68^Ga-DOTATOC and ^18^F-fluoride. However, BSREM reconstruction allowed for shorter acquisition than OSEM to reach similar noise levels for all tracers.

A note regarding the *β*-values should also be made. Our study uses a pilot version of the Q.Clear software, with the only difference to the commercially released software being the definition of *β*-values. Therefore, without affecting the results of our study, the evaluated *β*-values (originally set to 200–800 in steps of 200) were rescaled into 133–533 in steps of 133 to match the commercial version and to ensure reproducibility (i.e. *β*_pilot_ = 1.5 × *β*_commercial_).

We chose to compare the BSREM results to OSEM with 3 iterations 16 subsets, a 5-mm Gaussian post-processing filter, and PSF modelling, without further optimizing the OSEM algorithm. A narrower filter and a larger number of iterations would have increased the signal and simultaneously increased the level of noise. A previous study on ^18^F-FDG showed that a similar SNR and SBR was reached at higher noise levels for OSEM than for BSREM irrespective of filter width and number of iterations [6]. The OSEM algorithm is well established in the clinical routine and has previously been optimized and we therefore chose to apply the settings recommended by the manufacturer as our reference. Moreover, the BSREM algorithm incorporates PSF recovery, and consequently we chose to apply PSF also with OSEM to narrow down the number of variables, since the PSF models are the same in the two algorithms.

The use of BSREM reconstruction leads to an increased SUV_max_ and will potentially facilitate lesion detection but may also lead to false-positive results in the image reading, which needs to be assessed in comparative trials for the various PET tracers in clinical use. Also, for lesions that are assessed by applying uptake thresholds, such as in therapy monitoring of Hodgkin’s lymphoma by FDG PET whereby the lesion uptake is compared to that of the mediastinal blood pool and the normal liver tissue, the effect of BSREM on the result of the evaluation needs to be investigated. The same holds true for characterization of solitary lung nodules, which includes comparison of its FDG-uptake to that of the mediastinal blood pool.

## Conclusion

BSREM reconstruction of whole-body PET/CT examinations resulted in a tracer-dependent increase in tumour SUV_max_ as well as improved SNR and SBR with respect to conventional OSEM reconstruction, at equal acquisition times. Similar SNR, SBR, and noise levels could be attained with BSREM at relatively shorter acquisition times for matched administered dosages compared to OSEM. Alternatively, similar SNR, SBR, and noise levels could be attained with BSREM at lower administered dosages for matched acquisition times compared to OSEM. The findings of the present study thereby suggested a potential clinical benefit of BSREM over OSEM when applied for ^68^Ga-DOTATOC, ^11^C-acetate, and ^18^F-fluoride imaging.

## Supplementary information

**Additional file 1: Supplemental Figure S1.** Correlation between ^68^Ga-DOTATOC and ^18^F-FDG of noise in normal liver tissue (A), signal-to-noise ratio (B) and signal-to-background ratio (C) using BSREM reconstruction with β-values of 133, 267, 400 and 533. The data were normalized to those obtained by TOF OSEM (3 iterations, 16 subsets and 5-mm gaussian post-processing filter). **Supplemental Figure S2.** Correlation between ^18^F-fluoride and ^18^F-FDG of noise in normal liver tissue (A), signal-to-noise ratio (B) and signal-to-background ratio (C) using BSREM reconstruction with β-values of 133, 267, 400 and 533. The data were normalized to those obtained by TOF OSEM (3 iterations, 16 subsets and 5-mm gaussian post-processing filter). **Supplemental Figure S3.** Correlation between ^11^C-acetate and ^18^F-FDG of noise in normal liver tissue (A), signal-to-noise ratio (B) and signal-to-background ratio (C) using BSREM reconstruction with β-values of 133, 267, 400 and 533. The data were normalized to those obtained by TOF OSEM (3 iterations, 16 subsets and 5-mm gaussian post-processing filter).

## Data Availability

The datasets used and/or analysed during the current study are available from the corresponding author on reasonable request.

## Abbreviations

bp: Bed position
BSREM: Block-sequential regularized expectation maximization
CT: Computer tomography
FOV: Field of view
NEMA: National Electrical Manufacturers Association
OSEM: Ordered subsets expectation maximization
PET: Positron emission tomography
PSF: Point spread function
SBR: Signal-to-background ratio
SD: Standard deviation
SNR: Signal-to-noise ratio
SUV: Standardized uptake value
TOF: Time-of-flight
