# Supplementary material for: Evaluation of block-sequential regularized expectation maximization reconstruction of 68Ga-DOTATOC, 18F-fluoride, and 11C-acetate whole-body examinations acquired on a digital time-of-flight PET/CT scanner
Source: EJNMMI Phys. 2020 Jun 15;7:40. doi: 10.1186/s40658-020-00310-1 (PMC7295929; doi:10.1186/s40658-020-00310-1)
Supplement: Supplementary file 1 — Additional file 1: Supplemental Figure S1. Correlation between 68Ga-DOTATOC and 18F-FDG of noise in normal liver tissue (A), signal-to-noise ratio (B) and signal-to-background ratio (C) using BSREM reconstruction with β-values of 133, 267, 400 and 533. The data were normalized to those obtained by TOF OSEM (3 iterations, 16 subsets and 5-mm gaussian post-processing filter). Supplemental Figure S2. Correlation between 18F-fluoride and 18F-FDG of noise in normal liver tissue (A), signal-to-noise ratio (B) and signal-to-background ratio (C) using BSREM reconstruction with β-values of 133, 267, 400 and 533. The data were normalized to those obtained by TOF OSEM (3 iterations, 16 subsets and 5-mm gaussian post-processing filter). Supplemental Figure S3. Correlation between 11C-acetate and 18F-FDG of noise in normal liver tissue (A), signal-to-noise ratio (B) and signal-to-background ratio (C) using BSREM reconstruction with β-values of 133, 267, 400 and 533. The data were normalized to those obtained by TOF OSEM (3 iterations, 16 subsets and 5-mm gaussian post-processing filter). [file 40658_2020_310_MOESM1_ESM.docx]

**Evaluation of block-sequential regularized expectation maximization reconstruction of ^68^Ga-DOTATOC, ^11^C-acetate and ^18^F-fluoride whole-body examinations acquired on a digital time-of-flight PET/CT scanner**

# Elin Lindström^1,2^, Lars Lindsjö^3^, Anders Sundin^1^, Jens Sörensen^1,3^, Mark Lubberink^1,2^

^1^Radiology & Nuclear Medicine, Department of Surgical Sciences, Uppsala University, SE-751 85 Uppsala, Sweden; ^2^Medical Physics, Uppsala University Hospital, SE-751 85 Uppsala, Sweden; ^3^PET Centre, Uppsala University Hospital, SE-751 85 Uppsala, Sweden

Corresponding author: Elin Lindström; E-mail: elin.lindstrom@surgsci.uu.se


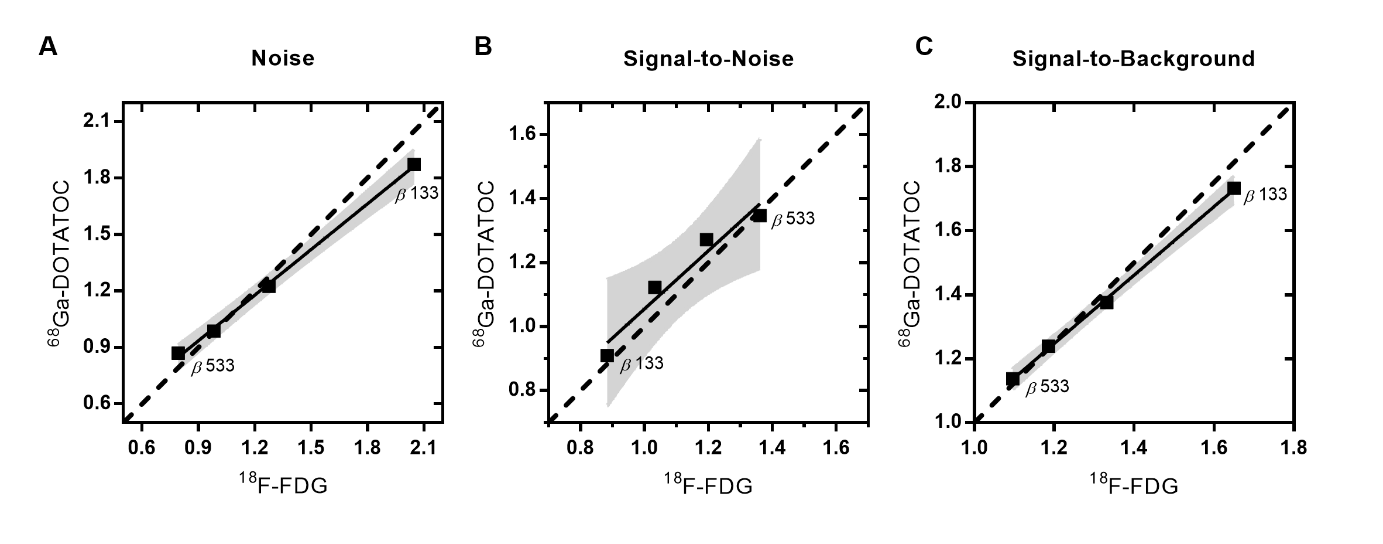


**SUPPLEMENTAL FIGURE 1.** Correlation between ^68^Ga-DOTATOC and ^18^F-FDG of noise in normal liver tissue (A), signal-to-noise ratio (B) and signal-to-background ratio (C) using BSREM reconstruction with β-values of 133, 267, 400 and 533. The data were normalized to those obtained by TOF OSEM (3 iterations, 16 subsets and 5-mm gaussian post-processing filter).


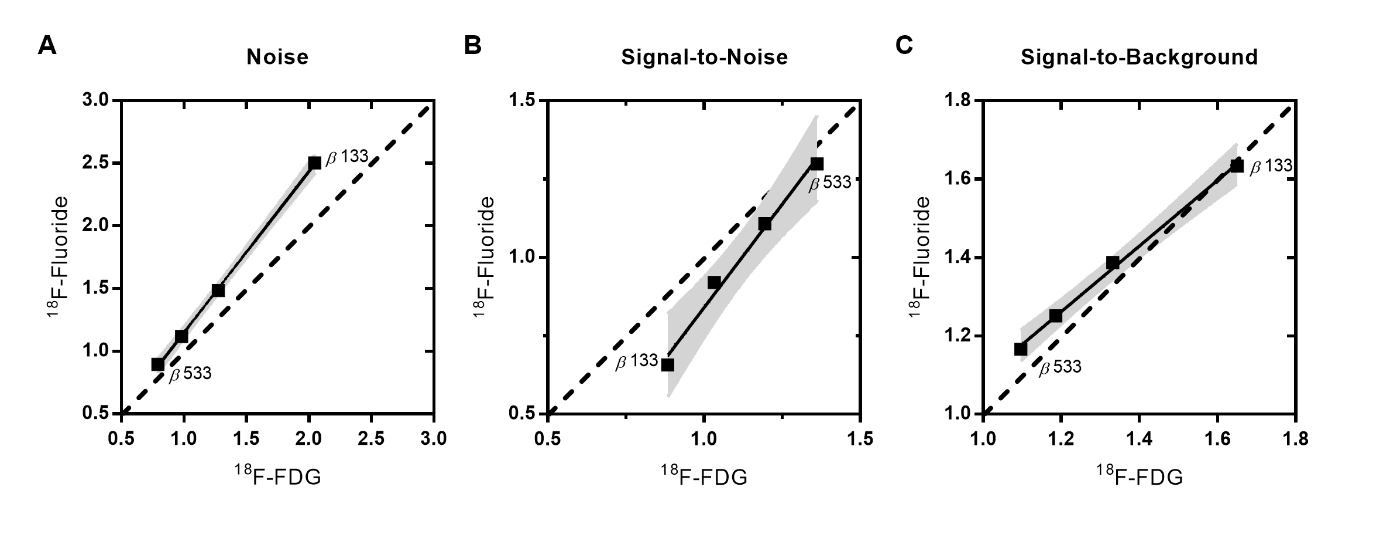


**SUPPLEMENTAL FIGURE 2.** Correlation between ^18^F-fluoride and ^18^F-FDG of noise in normal liver tissue (A), signal-to-noise ratio (B) and signal-to-background ratio (C) using BSREM reconstruction with β-values of 133, 267, 400 and 533. The data were normalized to those obtained by TOF OSEM (3 iterations, 16 subsets and 5-mm gaussian post-processing filter).


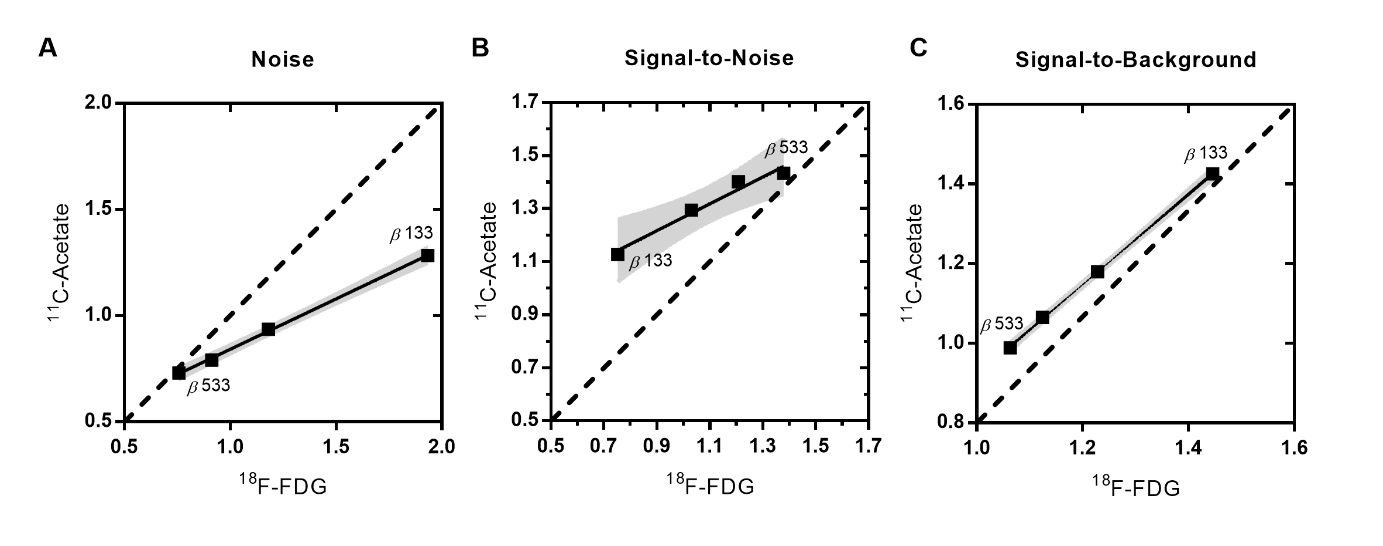


**SUPPLEMENTAL FIGURE 3.** Correlation between ^11^C-acetate and ^18^F-FDG of noise in normal liver tissue (A), signal-to-noise ratio (B) and signal-to-background ratio (C) using BSREM reconstruction with β-values of 133, 267, 400 and 533. The data were normalized to those obtained by TOF OSEM (3 iterations, 16 subsets and 5-mm gaussian post-processing filter).
